# Duchenne muscular dystrophy gene product expression is associated with survival in head and neck squamous cell carcinoma

**DOI:** 10.1038/s41598-025-94221-9

**Published:** 2025-03-28

**Authors:** Leanne Jones, Sonika Divakar, Lewis Collins, Wael Hamarneh, Phillip Ameerally, Karen Anthony, Lee Machado

**Affiliations:** 1 Centre for Physical Activity and Life Sciences, University of Northampton, University Drive, Northampton, NN1 5PH UK; 2 Department of Cancer and Genomic Sciences, College of Medicine and Health, University of Birmingham, Birmingham, UK; 3 Northampton General Hospital NHS Trust, Northampton, NN1 5BD UK; 4 Maxillofacial Department, Northampton General Hospital NHS Trust, Northampton, NN1 5BD UK

**Keywords:** DMD, Dystrophin, HNSCC, Cancer, Head, Neck cancer, Head and neck cancer, Cancer, Cell biology

## Abstract

Mutation of the Duchenne muscular dystrophy (DMD) gene causes neuromuscular disorders, but increasing evidence has implicated *DMD* in the development and progression of several major cancer types. This study investigates the prognostic and biological significance of *DMD* expression in head and neck squamous cell carcinoma (HNSCC). Analysis of The Cancer Genome Atlas (TCGA) data revealed that high *DMD* expression correlates with improved overall (median survival difference: 22 months, p = 0.0083) and progression-free (p = 0.0237) survival. The Dp71ab transcript is most strongly associated with better outcomes (median overall survival: 42 months, p = 0.0007). Notably, *DMD* expression levels stratify HPV-positive patients, identifying a *DMD* low/HPV-positive subgroup with poor outcomes. Immunohistochemical analysis of 50 HNSCC tissue cases confirmed dystrophin localisation in the nucleus and cytoplasm, with high nuclear expression linked to longer overall survival (mean difference: 31 months, p = 0.0497). Functional assays in HNSCC cells showed that Dp71ab overexpression disrupts nuclear morphology and reduces proliferation. Differential gene expression analysis additionally identified 388 upregulated and 30 downregulated genes, with pathways linked to muscle processes, ribosome biogenesis and non-coding RNA regulation. These findings highlight *DMD* as a potential biomarker and/or therapeutic target in HNSCC, warranting further mechanistic studies of Dp71 isoforms.

## Introduction

Mutations in the Duchenne muscular dystrophy gene, *DMD,* can lead to a dystrophinopathy; for example, frameshift mutations are causative for Duchenne muscular dystrophy (DMD) and associated with a severe and progressive muscle wasting phenotype^1^. The major (full-length) gene product is a 427 kDa rod-shaped dystrophin protein integral to muscle function; it associates with several plasma membrane proteins to form a dystrophin-associated protein complex (DAPC), a transmembrane structure connecting the actin cytoskeleton to the extracellular matrix^2^. The *DMD* gene also encodes additional dystrophin proteins (Dp, named by their size in kDa) which are differentially regulated by their own independent promotors in tissues other than muscle^1,3^. The most ubiquitous and predominant of these is Dp71 which undergoes alternative splicing to generate Dp71 isoforms with distinct subcellular localisations and functions^3^.

Interestingly, and in demonstration of an important role for the *DMD* gene beyond muscle function, growing evidence has implicated *DMD* in the development of all major cancer types with some studies specifically implicating Dp71 in tumorigenesis (reviewed by Jones et al.^4^). In 2017, Luce et al. undertook several tumour versus normal tissue comparisons finding that *DMD* ranked within the top 10% of differentially expressed genes^5^. In our own preliminary study on the effect of high versus low *DMD* gene expression on survival outcomes across all cancers, we found that high *DMD* expression might be associated with longer head and neck squamous cell carcinoma (HNSCC) survival times. Interestingly, this contrasts with other tumour types such as low-grade glioma (LGG) where our in-depth bioinformatic analysis revealed a detrimental effect of high *DMD* expression on survival^6^. Therefore, to further develop our understanding of the role of the *DMD* gene in cancer, and to highlight tissue-dependent effects, here we used HNSCC to exemplify tumours where high *DMD* expression appears protective.

HNSCCs can be derived from the epithelium of all main head and neck anatomical sites^7,8^. Symptoms vary depending upon the stage and anatomical location of the tumour, these include oral ulceration and pain, a sore throat, hoarseness of voice and a swelling or mass at the site of the tumour or in the neck^9^. As of 2019, head and neck cancer was the sixth most common worldwide with an incidence of more than 500,000 cases per year and approximately 380,000 deaths globally^10,11^. The five-year survival rate for HNSCC is approximately 50%, however, this depends on the anatomical site and stage of tumour on presentation; recurring HNSCC has a particularly poor prognosis with a survival rate of < 1 year^12–14^. Known risk factors include tobacco smoking, heavy alcohol consumption and human papillomavirus (HPV) status^15–17^. The genetic events and pathways that contribute to HNSCC pathogenesis remain poorly understood. Treatment is problematic and not always effective as detecting HNSCCs at an early intervention stage is challenging^18,19^. We studied the effect of high *DMD* gene and protein expression on survival outcomes and cellular functions across human HNSCC tissue and cell models.

## Results

### Low DMD expression is associated with poor survival outcomes in HNSCC

**Fig. 1:**
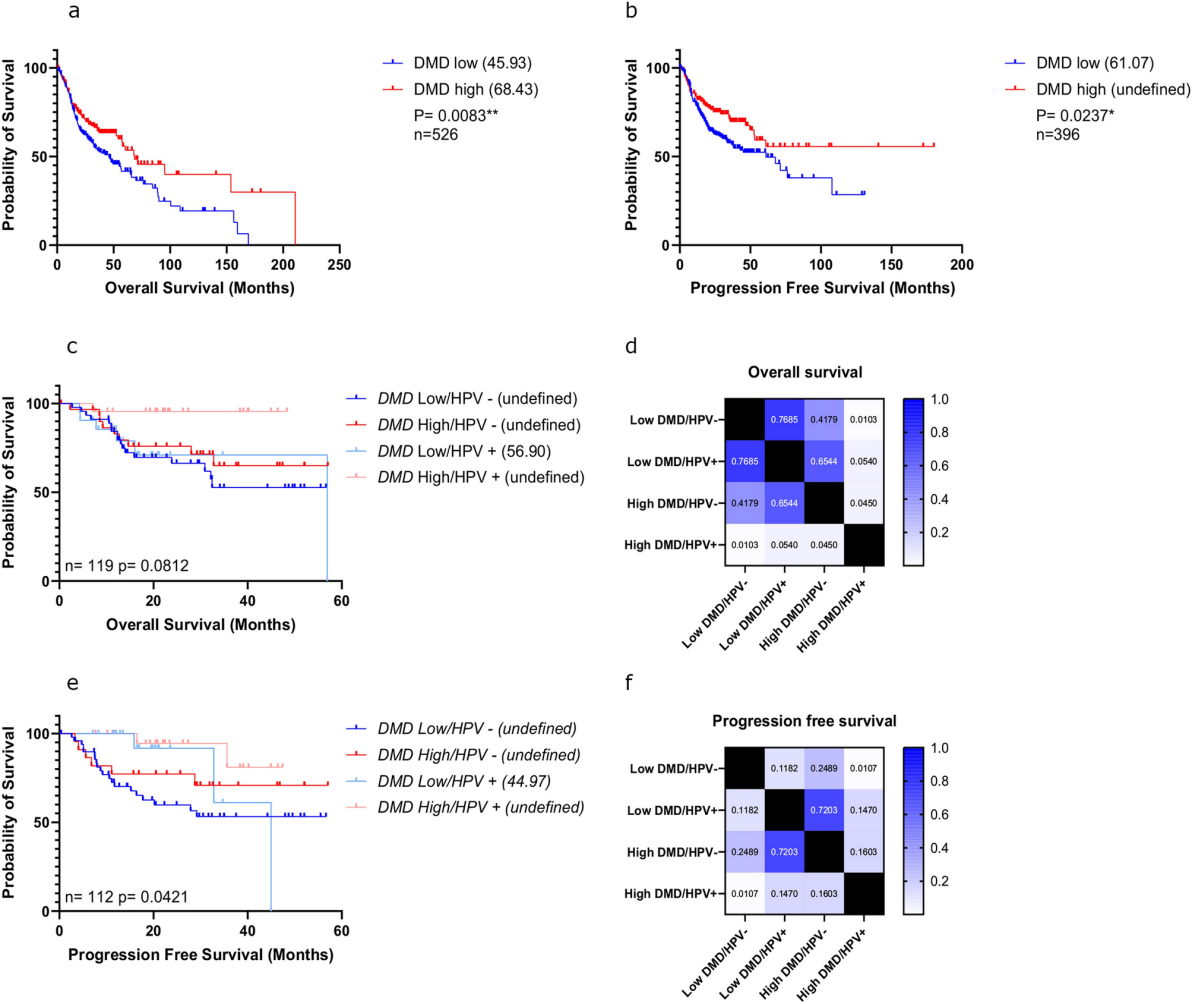
Kaplan Meier survival curves showing overall and progression free survival of HNSCC TCGA cases based on high/low *DMD* expression (**a**,**b**) or high/low DMD expression and HPV status (**c**–**f**). Median survival times are shown in brackets within the legends; n numbers and p values (determined using a log rank test) are indicated. Heat maps show the p values of each pairwise comparison for overall (**d**) and progression free (**f**) survival (Bonferroni adjusted alpha value for six comparisons = 0.008).

*In-silico* analysis was performed on a publicly available TCGA RNAseq dataset to determine whether there was an association between *DMD* expression and overall survival in HNSCC. A total of 526 cases were included (Supplementary Table 1, consisting of 117 cases from the larynx, 318 from oral cavity, 81 from oropharynx and ten from hypopharynx). Kaplan–Meier survival analysis identified significant associations for *DMD* expression with both overall (p = 0.0083) and progression free (p = 0.0237) survival (Fig. 1a,b). A median overall survival difference of approximately 22 months was observed between patients with high *DMD* expression compared to patients with low *DMD* expression. Thus, individuals with high *DMD* expression within HNSCC tumours survive longer than patients with low *DMD* expression. Given these findings, we next determined whether there were any survival differences when combining high/low *DMD* expression with human papillomavirus (HPV) status (an existing prognostic biomarker and diagnostic tool whereby a positive HPV status is beneficial for survival). Data were available for the oropharynx only and there was no difference in smoking intensity (pack years) between HPV positive and negative groups (data not shown). As expected, oropharynx individuals stratified into a HPV positive, high *DMD* category resulted in the best survival outcomes (Fig. 1c–f). HPV positive individuals with low *DMD* expression have a poorer overall survival compared to HPV positive, high *DMD* individuals, though this did not reach significance (Fig. 1d, pairwise p value = 0.054). These results indicate that *DMD* expression may further stratify HPV positive individuals into two subgroups, where those with low *DMD* expression have a worse outcome than what would be currently predicted in a clinical setting.

### Multiple individual DMD gene products are associated with HNSCC survival

**Fig. 2:**
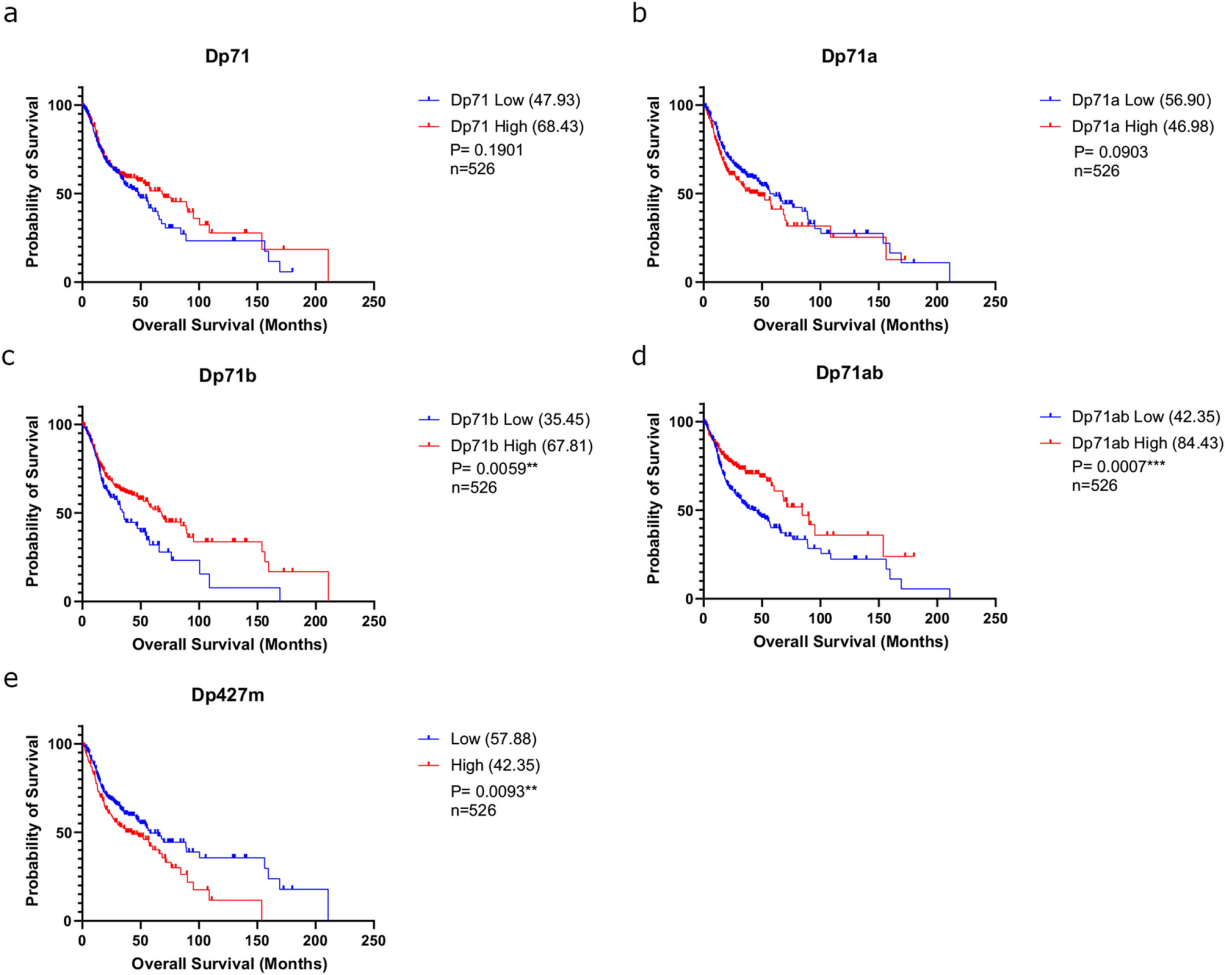
Kaplan Meier survival curves showing overall survival of HNSCC TCGA cases based on high/low *DMD* transcript expression. Median survival times are shown in brackets within the legends; n numbers and p values (determined using a log rank test) are indicated.

As the *DMD* gene produces multiple gene products further analyses were performed to see which are associated with HNSCC survival. First, we extracted RNAseq isoform data from the same TCGA cases analysed above. We plotted individual *DMD* transcript expression levels for all patients and determined that the four major Dp71 isoforms (Dp71, Dp71a, Dp71b and Dp71ab) are predominantly expressed in HNSCC (Supplementary Fig. 1). The full-length transcript, Dp427m, is also strongly expressed although this appears more variable than Dp71. We therefore next investigated the relationship between total *DMD* and individual transcript expression to give further clues as to which transcript(s) contribute to the survival associations described above. Spearman’s correlation analyses of total *DMD* against each individual transcript showed significant correlations between total *DMD* and Dp427 and all Dp71 transcripts across HNSCC patients (Supplementary Fig. 2). Dp71 transcripts were however more strongly correlated with total *DMD* expression suggesting that the survival differences observed between low and high *DMD* expression within HNSCC might be largely attributed to Dp71 expression. Additional Kaplan–Meier analyses were performed to see whether there was an overall survival difference in HNSCC when looking separately at the individual *DMD* gene variants. Significant associations between Dp71b (~ 32-month median survival difference, p = 0.0059), Dp71ab (~ 42-month median survival difference, p = 0.0007) and Dp427m (~ 15-month median survival difference, p = 0.0093) expression and overall survival in HNSCC were observed (Fig. 2). Of note, the survival trend observed for Dp427m was opposite to that of total *DMD*, Dp71b and Dp71ab expression. For Dp427m, high expression is associated with poorer survival. Overall, high total *DMD* and high Dp71 variant expression in HNSCC is associated with a better survival outcome and Dp71 variant expression is the most well correlated to total *DMD* expression within the TCGA HNSCC cohort. This trend was largely replicated by subgroup analysis on the larynx, oral cavity and oropharynx (subtype analysis was not performed on the hypopharynx due to low sample size), though significance was not maintained in all cases (Supplementary Fig. 3). Of note, Dp71ab remained significantly associated with survival (high expression beneficial) across two out of three subtypes (the oral cavity and oropharynx, Supplementary Fig. 3).

### Dystrophin protein is expressed in HNSCC tumours and tumour-derived cell lines

**Fig. 3:**
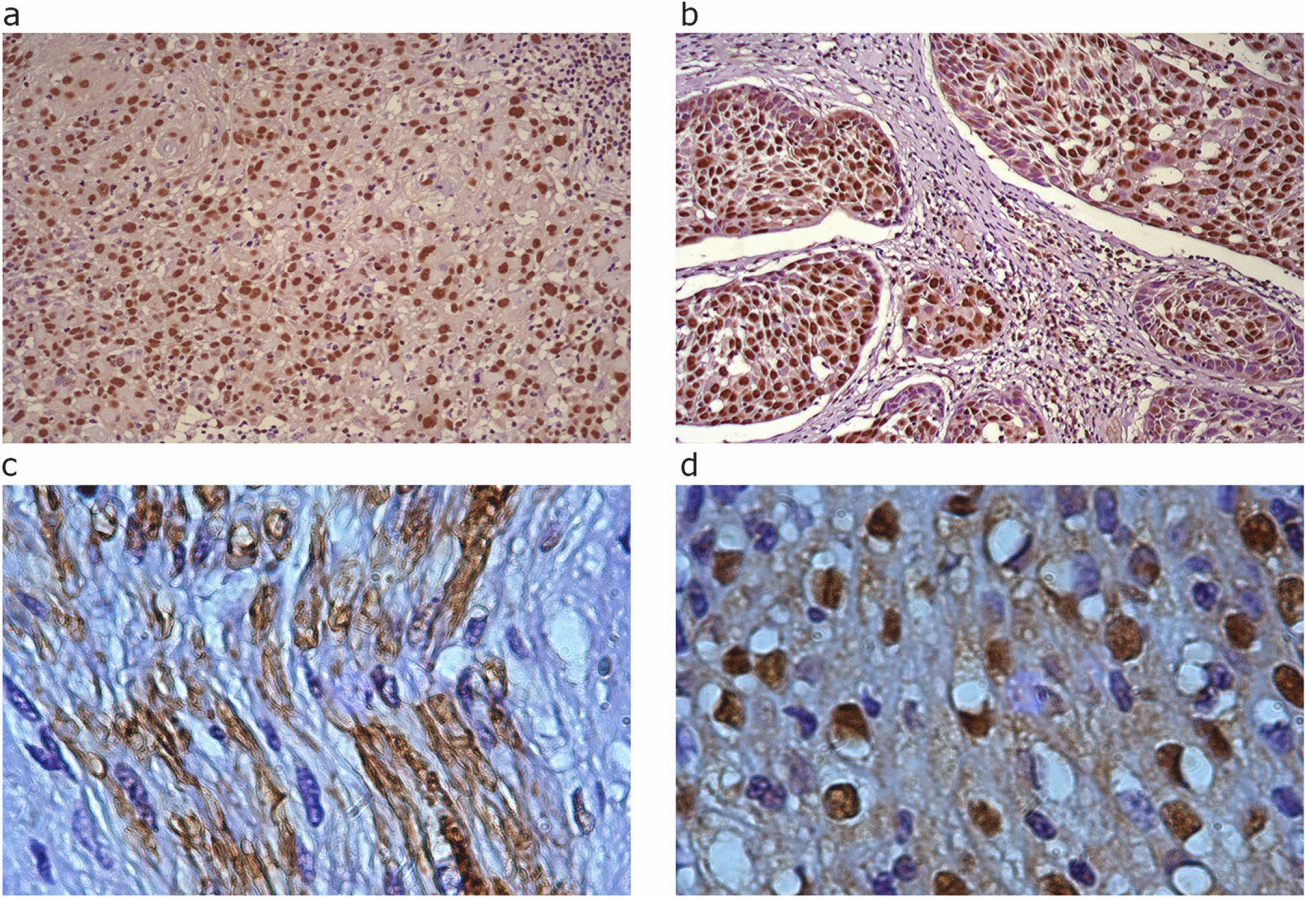
Representative images of positive immunohistochemical staining for dystrophin within tumour margins of the oral cavity (**a**,**c**) and oropharynx (**b**,**d**). Top panel images are × 40 and bottom panel × 100 magnification. Nuclear and cytoplasmic dystrophin staining is present in malignant squamous cells of the oral cavity and in malignant squamous cells within tumour nests of the oropharynx.

To characterise dystrophin protein expression in tumour tissue we examined 50 HNSCC tissue sections (Supplementary Table 2, comprising a 50:50 split of oral cavity and oropharynx cases). Tissue sections were stained with a dystrophin antibody specific to the C-terminus which theoretically detects all dystrophin proteins. Positive staining in the nucleus was observed in all but one case whilst cytoplasmic staining differed between anatomical subsites (Fig. 3). In the oral cavity, dystrophin staining was infrequently found in the cytoplasm and the staining was predominantly nuclear. Oropharyngeal tissue showed dystrophin staining in both the nuclei and cytoplasm of some cells and only nuclear staining in others. Dystrophin positive staining was distributed throughout both tumour and non-tumour tissue. The oral cavity had consistent dystrophin staining within the muscle, blood vessels, salivary glands, and nerve cells. Positive dystrophin staining was also consistently observed within lymphocytes and lobules of mucinous salivary glands across all oropharyngeal cases (Supplementary Fig. 4). These non-tumour structures served as an internal positive control for the staining.

**Fig. 4:**
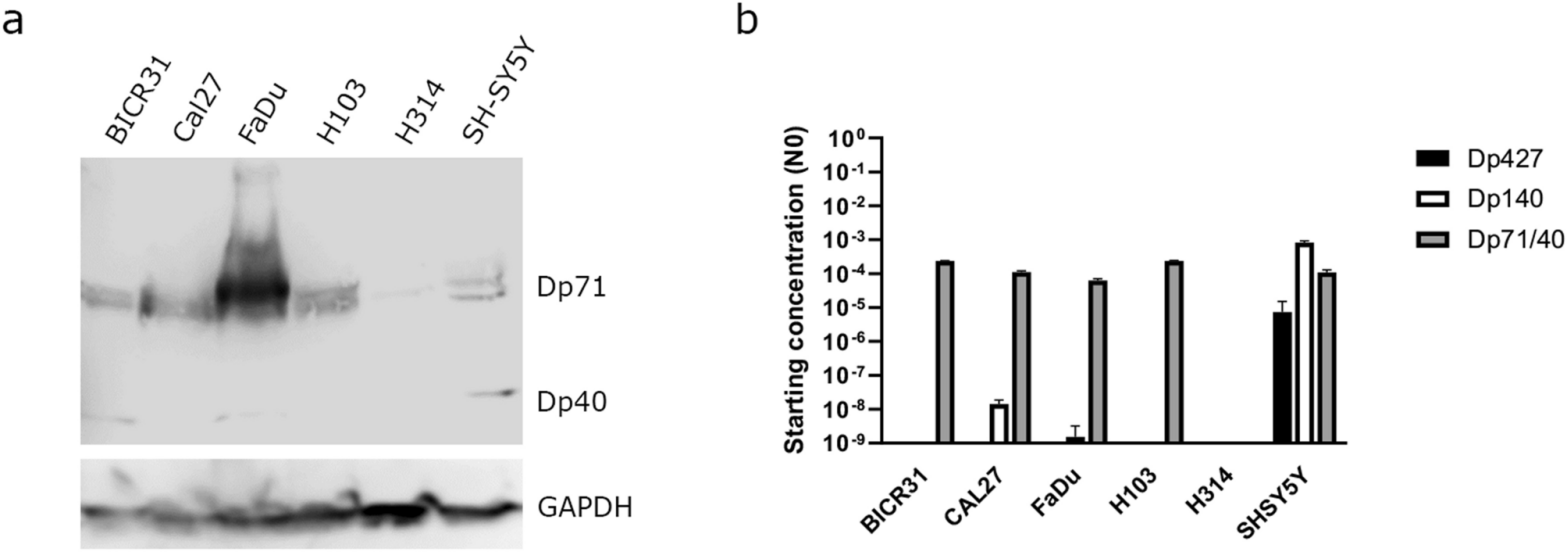
(**a**) Dystrophin protein expression across five HNSCC cell lines and a positive control (SH-SY5Y neuroblastoma); GAPDH was used as a loading control. Representative images of n = 3 (**b**) TaqMan qRT-PCR analysis of HNSCC cell lines; SH-SY5Y cells were used as a positive control. Data are presented on a log10 scale and represent the mean starting concentration (N0) ± SD from three biological repeats, each performed in triplicate. N0 units are arbitrary and normalised to the mean of 18S and GAPDH.

We next characterised a panel of five HNSCC cell lines for dystrophin expression by western blotting using the same C-terminal antibody (Fig. 4a). Dp71 appeared as a doublet (presumably representing splice isoforms) and was present in all cell lines except for H314 where it was extremely weak/absent. The higher of the two bands was very strongly expressed in the FaDu cell line. Weaker bands potentially corresponding to Dp40 expression were also consistently observed in two of the cell lines (BICR31 and FaDu). To further verify Dp71 expression, we performed qRT-PCR analysis using TaqMan probes specific to different dystrophin transcripts (Fig. 4b). The results confirmed the presence of Dp71 across all cell lines except H314. (Fig. 4b). Overall, and in-line with its known ubiquitous expression, these results suggest that Dp71 is the most predominant dystrophin transcript and protein within HNSCC tissue and highlight differences in Dp71 isoform composition (and subcellular localisation) between different subsites of head and neck tumours.

### Dystrophin protein expression is associated with poor survival in HNSCC

**Fig. 5:**
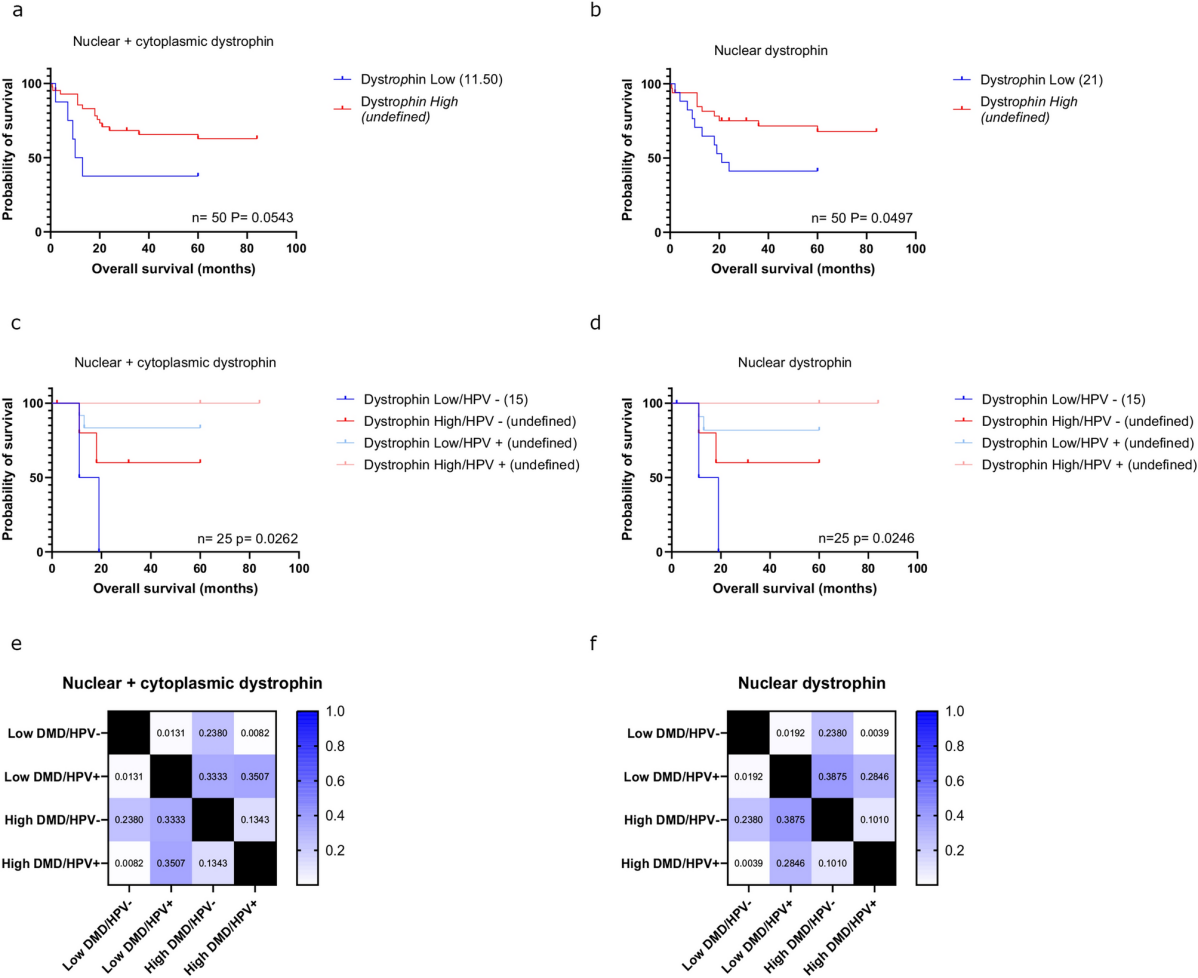
Kaplan Meier survival curves showing overall survival of a HNSCC tissue cohort (n = 50) based on high/low whole cell dystrophin expression (**a**), high/low nuclear dystrophin expression (**b**), high/low whole cell dystrophin and HPV status (**c**) and high/low nuclear dystrophin and HPV status (**d**). HPV status was analysed only for the oropharyngeal cases (n = 25). Median survival times are shown in brackets within the legends; n numbers and p values (determined using a log rank test) are indicated. Heat maps show the p values of each pairwise comparison for whole cell (**e**) and nuclear (**f**) dystrophin expression (Bonferroni adjusted alpha value for six comparisons = 0.008).

Since we confirmed the expression of dystrophin protein within HNSCC tumour tissue, we next sought to determine whether low dystrophin protein expression was linked to poor survival as was observed with the TCGA RNAseq dataset above. Kaplan–Meier survival analysis shows a clear separation in survival curves whereby low dystrophin expression is associated with a poor overall survival (Fig. 5). This was significant when examining specifically nuclear dystrophin protein expression (a mean survival difference of 31 months, p = 0.0497, Fig. 5b). Thus, these results from a small cohort of only 50 cases effectively replicate the in-silico mRNA expression analysis conducted above and additionally implicate nuclear dystrophin involvement. We next repeated the analysis of dystrophin expression alongside HPV status (Fig. 5c–f). Since HPV is only a relevant biomarker for oropharyngeal tumours and not those of the oral cavity, we performed this analysis on the 25 cases derived from the oropharynx. As expected, and predicted from our prior analysis, there was a significant difference in overall survival outcomes across the different groups (Fig. 5c,d). Individuals who had low nuclear dystrophin expression and were HPV negative had a particularly poor overall survival (median survival of 15 months). Conversely, the other groups had survival probabilities that did not decrease below 50% over 100 months of observations. HPV positive individuals had the best survival outcomes, but when dichotomised by low versus high dystrophin expression, the low dystrophin group appears to have a poorer survival than what would have been predicted by HPV status alone. In summary, individuals who are HPV negative may particularly benefit from being further stratified using dystrophin expression status.

### HNSCC cell lines overexpressing Dp71ab have altered nuclear morphology and a reduction in cellular proliferation

**Fig. 6:**
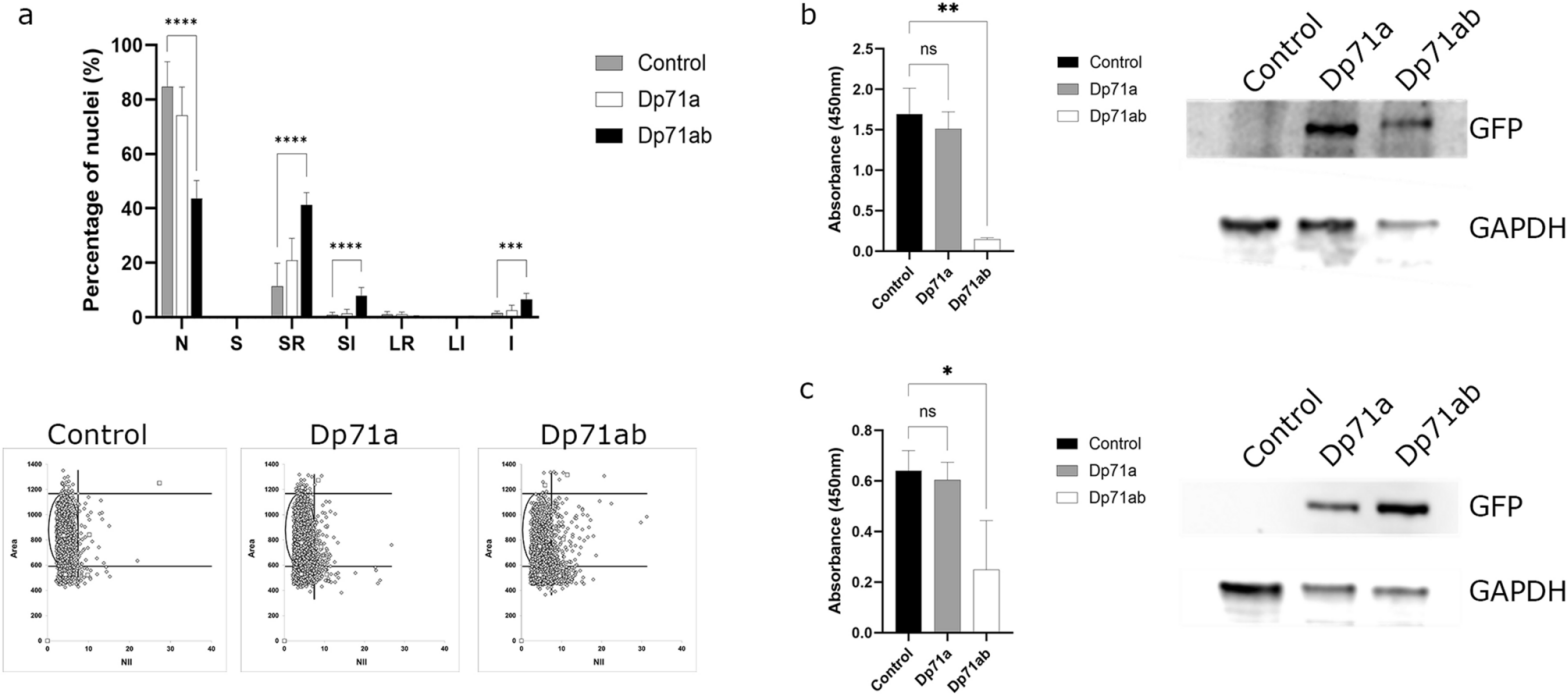
(**a**) Nuclear morphology analysis of H314 cells overexpressing GFP-Dp71a or GFP-Dp71ab. N = normal, S = small, SR = small regular, SI = small irregular, LR = large regular, LI = large irregular and I = irregular. The bar chart plots mean percentages of nuclei in each group ± SD; scatter graphs show the area versus nuclear irregular index (NII) of each individual nuclei. (**b**,**c**) WST-1 assay measuring cell metabolic activity at 450 nm absorbance (mean ± SD), 48 h post transfection in FaDu (**b**) and H314 (**c**) cells. Representative western blots show successful transfection determined using an anti-GFP antibody and GAPDH as a loading control. All data are n = 3 performed with a mock transfection control. P values were determined using a one-way ANOVA and Dunnett’s multiple comparisons test, ns symbolises no significance.

The above findings suggest that high Dp71 expression may be tumour suppressive. However, we cannot attribute these results to a specific Dp71 isoform(s). Given that Dp71a (-71, + 78) was not significantly associated with HNSCC survival, but Dp71b (+ 71, -78) and Dp71ab (-71, -78) were, we hypothesised that the loss of exon 78 may be responsible for the tumour suppressive effect of Dp71 in HNSCC. To test this, we therefore studied the effect of overexpressing GFP-tagged Dp71a and Dp71ab in both H314 (oral cavity, no endogenous Dp71 expression) and FaDu (hypopharyngeal, high endogenous Dp71 expression) cell lines on cell migration and proliferation. No significant differences were observed in cell migration (2D scratch and 3D spheroid assays, Supplementary Figs. 5 and 6). However, we noticed differences in the nuclear morphology of Dp71ab transfected H314 cells which prompted us to quantify nuclear morphology using a nuclear irregularity index tool (Fig. 6a). No significant differences in nuclear morphology were found in the FaDu cell line, however Dp71ab transfected H314 cells had a significantly reduced number of normal nuclei compared to control (85% versus 44%, p =  < 0.0001). This was accompanied by a significant increase in small regular (p =  < 0.0001), small irregular (< 0.0001) and irregular (p = 0.0005) nuclei. This is suggestive of an increased proportion of cells that are undergoing apoptosis and/or mitotic damage or catastrophe. To explore this further we conducted a proliferation/metabolic activity (WST-1) assay. With both the H314 and FaDu cell lines, the results show a significant reduction in metabolic activity in cells transfected with Dp71ab compared to untransfected control cells (Fig. 6b,c). These findings complement the nuclear morphology analysis suggesting that mitosis is affected by Dp71ab overexpression. We therefore next examined the Ki67 proliferative index of our HNSCC tissue cohort to determine whether cases with high dystrophin expression have a lower proliferative index than cases with low dystrophin levels (Supplementary Fig. 7). However, we did not find a significant difference in the Ki67 proliferative index between high and low dystrophin expressing groups. Though we note our use of a pan dystrophin protein antibody for immunohistochemistry cannot be directly compared to the overexpression of specific Dp71 isoforms in cell lines.

### Differential gene expression and pathway analysis of high versus low *DMD* expression

**Fig. 7:**
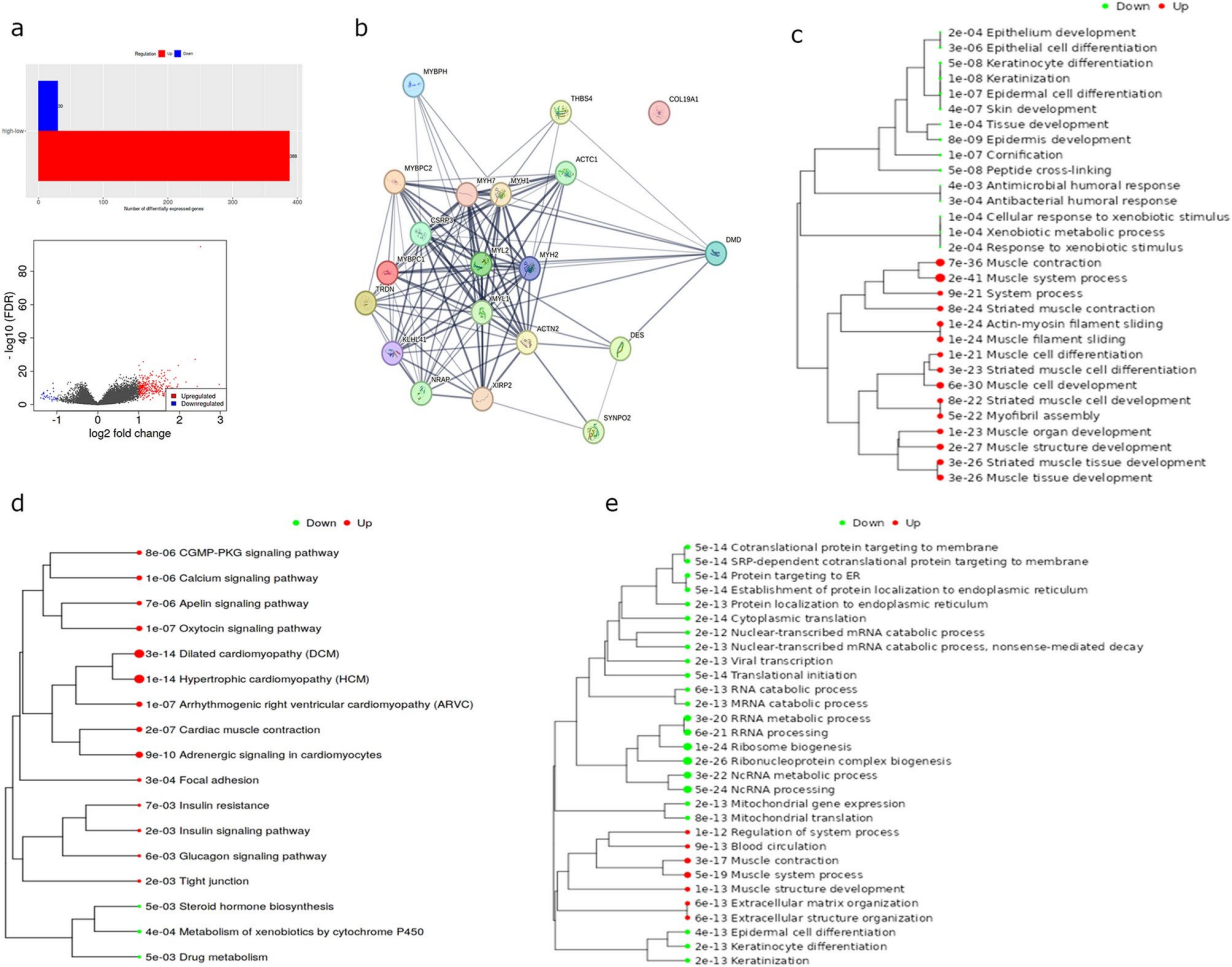
(**a**) Bar and volcano plots showing the number of identified DEGs in HNSCC TCGA cases with high vs low *DMD* expression. (**b**) STRING protein–protein interaction network of the top 20 upregulated DEGs. Line thickness depicts confidence between interactions. PPI enrichment p-value =  < 1.0e-16, number of edges = 109, expected number of edges = 2. GO Biological Processes (**c**) and KEGG^44^ (**d**) enrichment trees of the up- and downregulated DEGs. (**e**) Pathway analysis (GO Biological Processes) showing enriched pathways in high versus low *DMD* cases. Dot size indicates adjusted p value.

To aid future investigation into the functional role(s) of the *DMD* gene in HNSCC tumourigenesis we undertook a detailed bioinformatic analysis of the DEGs in the TCGA cases with high versus low *DMD* expression. We used an integrated web application, iDEP, for data pre-processing and to identify the DEGs (DESeq2 method). A total of 418 DEGs were identified. These included 30 down-regulated genes and 388 up-regulated genes (FDR cut off 0.01, minimum fold change 2, Fig. 7a). The Search Tool for the Retrieval of Interacting Genes (STRING) was used to identify the protein–protein interaction (PPI) network connectivity of the top 20 upregulated genes (Fig. 7b). The PPI enrichment P-value was < 1.0e − 16 using a median confidence score of 0.4 and the number of edges was 109. The expected number of edges for a random set of proteins of similar size was 2 strongly suggesting functional intersection of the identified DEGs. Proteins associated with muscle structure and cardiac function were overrepresented within the STRING network. To further examine the functional annotation of the DEGs we used the enrichment analysis (gene ontology [GO] biological processes and Kyoto Encyclopedia of Genes and Genomes [KEGG]) for DEGs tool in iDEP (Fig. 7c,d). The up-regulated genes are enriched in processes relating to muscular systems; the down-regulated genes are enriched in epithelial skin and tissue development and differentiation. The most significant result returned by the KEGG annotation was an up-regulation of the dilated cardiomyopathy and hypertrophic cardiomyopathy pathways. We next performed a separate pathway analysis using the fold-change values of all the genes in our dataset to identify coherently altered pathways upon high vs low *DMD* expression. We used the generally applicable gene set enrichment (GAGE) method within iDEP and the genes were annotated according to GO biological processes (Fig. 7e). Pathways relating to ribosome biogenesis and non-coding RNA processing were downregulated whilst pathways relating to muscle systems were upregulated.

## Discussion

Our results show high *DMD* expression is associated with better overall HNSCC survival and that this effect may be largely attributed to the expression of Dp71ab. We recently reviewed the literature surrounding the involvement of *DMD* across all major cancer types, including HNSCC^4^. Whilst it is clear there are opposing effects on *DMD* expression depending on tumour or tissue type (e.g. we previously demonstrated that high *DMD* expression in low-grade glioma is linked to poor survival^6^ in contrast to this work on HNSCC), our results are largely in agreement with prior literature examining *DMD* in HNSCC. One study identified that the *DMD* single nucleotide polymorphism (SNP) rs5927056 is associated with a reduced risk of nasopharyngeal carcinoma^20^. Another study revealed *DMD* as one of several large common fragile site (CFS) genes to be significantly downregulated in oropharyngeal squamous cell carcinoma (OPSCC); collectively the decreased expression of these genes is associated with an increased occurrence of OPSCC^21^. Thus, it seems that maintaining high *DMD* expression across multiple subtypes of HNSCC is protective. Alnassar et al. also recently demonstrated that the downregulation of dystrophin expression is linked to reduced survival across diverse tumours including HNSCC^22^. Most studies, however, do not consider the full complexity of dystrophin and its multiple proteins and isoforms thereof. It is important that future work in this area determine the composition of dystrophin proteins (and the dystrophin-associated protein complex, DAPC) within the tissue or cells of interest given known differences^3^. Indeed, a limitation of the current work is that we were unable to determine (due to both tissue and isoform specific antibody availability) the exact dystrophin species we detected by immunohistochemistry. For example, we used a rabbit polyclonal dystrophin antibody lacking publicly available epitope mapping information. However, assessing our findings in the context of prior literature^4,23–28^, the ubiquitous nature of Dp71 and our use of a C-terminal dystrophin antibody we are confident that Dp71 is of significant interest in tumourigenesis. Indeed, other proteins such as p63 also act as a tumour suppressor or oncogene in different contexts. Full length TAp63 isoforms have tumour-suppressor functions while N-terminally truncated ΔNp63 isoforms have oncogenic properties^29–31^, this is strikingly similar to what we observe with different dystrophin protein products.

Our work has for the first time evidenced the localisation of dystrophin in both the nucleus and cytoplasm of malignant oral cavity and oropharynx tissues with more predominant nuclear expression in the oral cavity. Immunohistochemical staining in a small pilot study involving tissue from 50 patients showed high intensity nuclear dystrophin staining was significantly associated with a better overall patient survival. This result is particularly striking given we had tissue from only 50 individuals, further analysis using a larger tissue microarray cohort would be beneficial to replicate these findings for dystrophin as an independent prognostic marker in HNSCC. HPV status remains the only clinically validated biomarker of survival for HNSCC^32^. We show that low *DMD* gene and/or dystrophin protein expression further stratified HPV positive individuals (known good prognosis) into a new subgroup with a poorer overall survival outcome than predicted by HPV status alone. We are unclear whether this data is confounded by smoking status as the number of cases with available HPV and smoking status was too small to address sufficiently. Future analysis is needed to confirm the clinical relevance and definitively evidence *DMD* gene and/or dystrophin protein expression as a useful diagnostic and prognostic tool alongside HPV status.

Nonetheless, our functional work confirms dystrophin biology is of interest regardless of diagnostic value. We revealed the biological pathways and cellular processes associated with high *DMD* expression, and interestingly, the downregulation of genes involved in ribosome biogenesis and non-coding RNA processing was also observed when comparing high versus low *DMD* expression in LGG^6^. More work is needed to fully explore these RNAseq findings including whether the upregulated genes involved in muscle processes is pertinent to HNSCC tumorigenesis or a consequence of the muscle-rich environment of these tumours. For example, the upper oesophagus is primarily composed of striated muscle where high Dp427 could be a biomarker for normal tissue. We showed that Dp71ab overexpression perturbs nuclear morphology and significantly reduces proliferation in HNSCC cell lines. Known nuclear roles of Dp71 (reviewed in Naidoo et al.) include nuclear matrix and envelope scaffolding^33–35^, DNA damage^25^ and cell division^36^ all of which are implicated in cancer. We previously proposed that the ratio of full-length dystrophin (Dp427) to Dp71 expression may be important in neoplastic progression^4^; it is also possible that rapid mitotic cycles may not accommodate the sustained expression of Dp427 (given its lengthy transcription time), potentially contributing to its selective loss and downregulation in aggressive cancers. Considering our findings described here, for HNSCC, we can suggest that Dp71ab is associated with HNSCC survival via altered cell proliferation.

No studies have yet ascertained whether the *DMD* gene plays a driving role in cancer or whether findings such as ours are passenger effects. This remains an important question given the potential prognostic utility and improved risk stratification across multiple tumours and the drug target potential of *DMD* gene products such as Dp71. Padder et. al. recently developed a mathematical model to simulate the interactions between dystrophin and components of the tumour micro-environment to predict how changes in dystrophin levels affect tumour growth and progression. Their findings support a key role for dystrophin expression in influencing factors like tumour size and spread^37^. Overall, this work has for the first time identified a role for *DMD*, and specifically Dp71ab, expression in the tumorigeneses of HNSCC. The use of *DMD* gene and/or dystrophin protein expression in combination with HPV status may add prognostic value and define additional subgroups that may better tailor treatment decisions. This work adds to the accumulating evidence for an important role of dystrophin in cancer which warrants further investigation in more complex disease models.

## Materials and methods

### Clinical datasets

The Cancer Genome Atlas (TCGA) dataset and corresponding clinical data was downloaded from cBioPortal querying for the *DMD* gene (Head and Neck Squamous Cell Carcinoma, TCGA, Firehose Legacy). Data extracted was mRNA expression, RSEM (batch normalized from Illumina HiSeq_RNASeqV2). The Firebrowse portal was used to extract TCGA RNAseq isoform data from the HNSC mRNASeq archives (illuminahiseq_rnaseqv2-RSEM_isoforms_normalized MD5) and case IDs matched to those from cBioPortal. *DMD* transcript IDs were matched to specific transcripts using the table browser tool from UCSC genome browser.

### Survival analysis

X-tile (version 3.6.1, Yale University) was used to dichotomise the datasets into high and low *DMD* (or individual gene product) expression groups using a minimal P-value approach. The optimal cut-point (the brightest pixel on the X-tile plot of chi squared log-rank values) generated by X-tile was used for survival analysis. Cut-points were generated for each gene product and used across all tumour subtypes. The RSEM cut-point used for the TCGA total *DMD* expression dataset was 287.89. The cut-points for Dp427m, Dp71, Dp71a, Dp71b and Dp71ab were 21, 2.55, 12.5, 20.32 and 160 respectfully. Kaplan–Meier curves were analysed using the log-rank test in GraphPad. Hypopharynx data was included in the overall analyses but due to containing only ten patients was omitted from subset analyses. Age at diagnosis data was split into young and old groups using the median (67.5 years of age). Unless otherwise stated, significance was set at 0.05 and asterisks used to indicate the level of significance: *P =  ≤ 0.05, **P =  ≤ 0.01, ***P =  ≤ 0.001 and ****P =  ≤ 0.0001. For multiple pairwise comparisons the alpha value was adjusted and indicated where relevant.

### Immunohistochemistry

Formalin-fixed paraffin-embedded sections from 50 HNSCC cases were obtained from King’s Health Partners Cancer BioBank (HTA Licence No: 12121, REC No: 12-EE-0493). The cohort included 25 oral cavity cases and 25 oropharynx cases. Sections were deparaffinised with xylene and rehydrated through graded alcohol. Antigen retrieval was achieved by microwave heating in pH 6.0 citrate buffer. A C-terminal anti-dystrophin antibody (Abcam, Ab15277, 1:100) and/or anti-Ki67 (Abnova, Mki67 PAB12127, 1:500) followed by a Tyramide SuperBoost™ kit (ThermoFisher Scientific, Paisley, U.K.) were used according to manufactures’ instructions. Staining was visualised using 3,3′-diaminobenzidine tetra hydrochloride (DAB), and counterstained with haematoxylin. Whole cell and nuclear dystrophin expression was analysed using the semi-quantitative H-score method categorising staining as no staining, weak, moderate and strong staining. Mean H-score values from two independent scorers were calculated and agreement confirmed by intraclass correlation analysis. Mean H-scores were dichotomised into high and low dystrophin expression using X-tile. The H-score cut-points for whole cell overall survival, whole cell progression free survival, nuclear overall survival and nuclear progression free survival were 29, 95, 40 and 40 respectively. HPV data was only available for the oropharynx cohort so cutpoints were generated using the median nuclear and whole cell H score values (112.7 and 127 respectively).

The percentage of Ki67 positive cells (Ki67 proliferative index) was calculated blind to knowledge of dystrophin expression using whole slide scanned MRXS images and the QuPath (v0.5.1) positive cell detection tool. Oral cavity and oropharynx training sets containing areas manually classified as tumour, stroma or immune cells were applied to entire images providing Ki67 scores from within tumour areas only. These were split into high and low *DMD* groups as above.

### Cell culture

All cell lines were a kind gift from Professor Paul Murray at the University of Birmingham, U.K. BICR31 (tongue squamous carcinoma), H314 (floor of mouth squamous cell carcinoma), H103 (tongue squamous cell carcinoma), Cal27 (tongue squamous cell carcinoma) and FaDu (hypopharyngeal squamous cell carcinoma) were cultured in DMEM with 2 mM glutamine and either 10% (H314, H103, Cal 27 and FaDu) or 5% (BICR 31) foetal bovine serum at 37 °C at 5% CO_2_. BICR31, H314 and H103 were supplemented with 0.5 µg/ml hydrocortisone. Transfections were performed in six well plates in serum-free media (Opti-MEM™) using 2.5 µg of plasmid DNA and Lipofectamine 2000 (ThermoFisher Scientific) as per manufacturer’s instructions. Green fluorescent protein (GFP)-Dp71a and GFP-Dp71ab plasmid constructs were a kind gift^38^.

### Quantitative reverse transcription polymerase chain reaction (RT-qPCR)

Total RNA was extracted using Trizol. RNA was DNase treated using an RNase-free DNase kit (Qiagen, Manchester, U.K.) and reverse transcription performed with 0.5 µg RNA and random hexamers using the RevertAid H minus first strand cDNA synthesis kit (ThermoFisher Scientific). cDNA was diluted 1:5 with nuclease free water and 50 ng used in triplicate for qPCR using the TaqMan Fast Advanced Master Mix and the StepOnePlus™ real-time PCR system (Applied Biosystems). TaqMan FAM-MGB assays (ThermoFisher Scientific) specific for Dp427 (exon 18–19 boundary, assay ID Hs01049416_m1), Dp140 (custom designed, assay ID APEPZC3) or Dp71/40 (assay ID: Hs01049405_m1) expression were used. Raw fluorescence data (RDML) was exported and analysed using RDML-LinRegPCR web-based tool (version 1.8.1 https://www.gear-genomics.com/rdml-tools/linregpcr.html) which provides an efficiency corrected starting concentration (N0) from raw fluorescence values^39^. Data was normalised to the mean of 18S (Hs99999901_s1) and GAPDH (Hs02758991_g1).

### Western blotting

Protein lysates were generated by scraping directly in boiling SDS sample buffer (50 µl/well). SDS–polyacrylamide gel electrophoresis (PAGE) was performed using 20 µg of protein and 10% Bis–Tris NuPAGE polyacrylamide gels (Bio-Rad Laboratories). Proteins were transferred onto PVDF membranes using the Bio-Rad Mini Protean Tetra Cell wet transfer system at 100 V for one hour on ice. Post transfer, membranes were blocked in 5% non-fat milk in PBS-T, washed and incubated overnight at 4 °C with primary antibodies to dystrophin (Abcam ab15277, 1:500), GFP (Invitrogen, G10362, 1:200) or GAPDH (Santa Cruz, sc-365062, 1:2000) diluted in blocking buffer. Membranes were washed and incubated with secondary anti-mouse or anti-rabbit HRP antibodies (Dako; 1:2000 and 1:1500 respectively) for one hour at room temperature. Proteins were visualised using ECL Plus (ThermoFisher Scientific).

### Functional cell assays

Image J (version 1.54) was used to analyse nuclear morphology using the nuclear morphometric analysis (NMA) plugin^40^. Ten random images per cell condition (minimum of 300 nuclei) stained with NucBlue (× 20 magnification) were analysed. Scratch assays were performed using a p200 pipette tip on confluent cells serum-starved (16 h) in Opti-MEM™. The cell free area was measured using the Image J MRI Wound Healing Tool (RRID:SCR_025260, version 1.28 https://github.com/MontpellierRessourcesImagerie/imagej_macros_and_scripts/wiki/Wound-Healing-Tool). Spheroid assays (hanging drop method) were performed as described elsewhere^41^ and total spheroid area measured using the SpheroidJ plugin tool for image J (version 1.0.1 https://github.com/joheras/SpheroidJ)^42^. Proliferation was measured using a water-soluble tetrazolium salt (WST-1, Abcam) assay as per manufacturer’s instructions. Plates were read using a Promega GloMax platereader at 450 nm with a reference wavelength of 600 nm. All statistical analyses were performed using GraphPad Prism (version 10.2.3 https://www.graphpad.com/; one-way ANOVA with Dunnett’s multiple comparisons test, alpha set to 0.05).

### Differential expression and pathway analysis

The integrated web application, iDEP 0.9330, hosted at http://ge-lab.org/idep/ was used for data pre-processing and log transformation of normalised expression values formatted into low and high dystrophin groups using the cut-points derived from X-tile^43^. Volcano plots and enrichment trees were produced in iDEP. Differentially expressed gene (DEG) pathways were produced using the DESeq2 method in iDEP with a false discovery rate (FDR) cut-off of 0.05 and a minimum fold-change of 2. STRING analysis was conducted through the enrichment DEG2 tab in iDEP 0.9330, hosted at http://ge-lab.org/idep/ with the protein–protein interaction (PPI) network of DEGs set to 20 with an FDR cutoff of 0.01.

## Supplementary Information

Supplementary Information.

## Data Availability

Pre-existing data underpinning this publication are openly available from cBioPortal at https://www.cbioportal.org/ (Head and Neck Squamous Cell Carcinoma, TCGA, Firehose Legacy).
