# Supplementary material for: Duchenne muscular dystrophy gene product expression is associated with survival in head and neck squamous cell carcinoma
Source: Sci Rep. 2025 Mar 28;15:10754. doi: 10.1038/s41598-025-94221-9 (PMC11953289; doi:10.1038/s41598-025-94221-9)
Supplement: Supplementary file 1 — Supplementary Information. [file 41598_2025_94221_MOESM1_ESM.docx]

**Supplementary table 1.** Summary of clinical variables within the HNSCC TCGA dataset

**
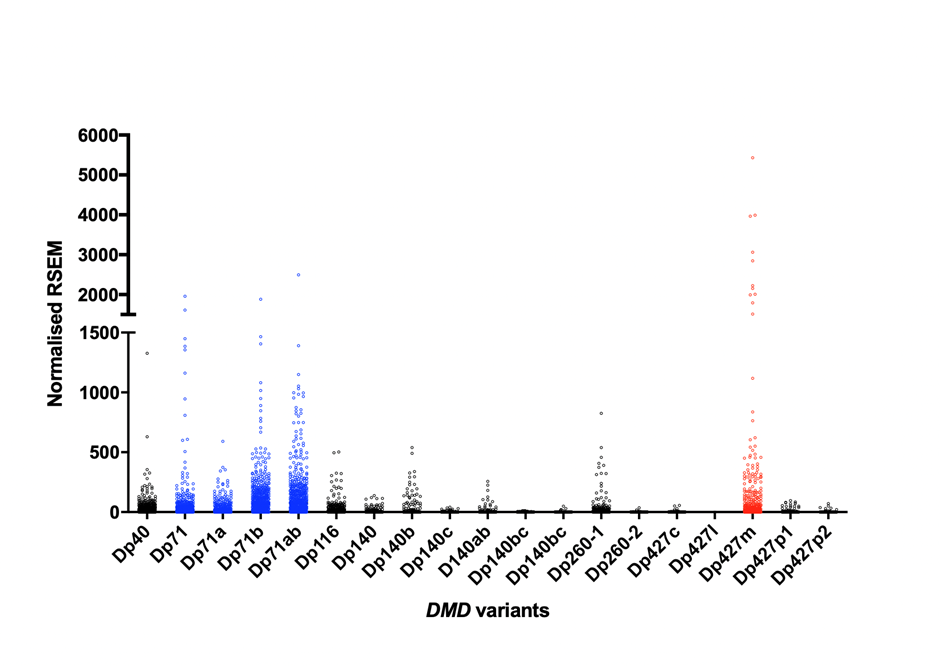
**

**Supplementary figure 1.** mRNA-seq expression of *DMD* gene variants and their isoforms in primary HNSCC tissue. Each data point represents an individual tissue sample and its representative mRNA expression levels within primary HNSCC tissue.

**
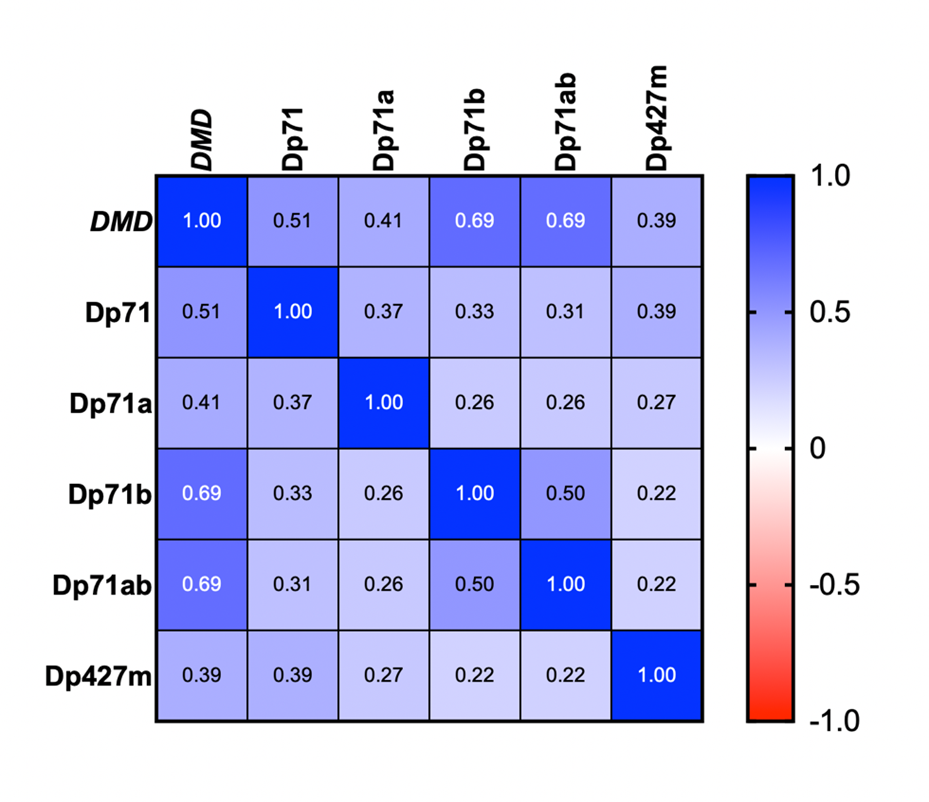
**

**Supplementary figure 2.** Spearman’s correlation analyses between total *DMD* and individual gene product expression within the overall HNSCC TCGA cohort (n=526). Legend indicates Spearman’s Rho values. Deep blue shown as 1 in the legend indicates a very strong positive correlation and deep red shown as -1 depicts a very weak negative correlation.

**
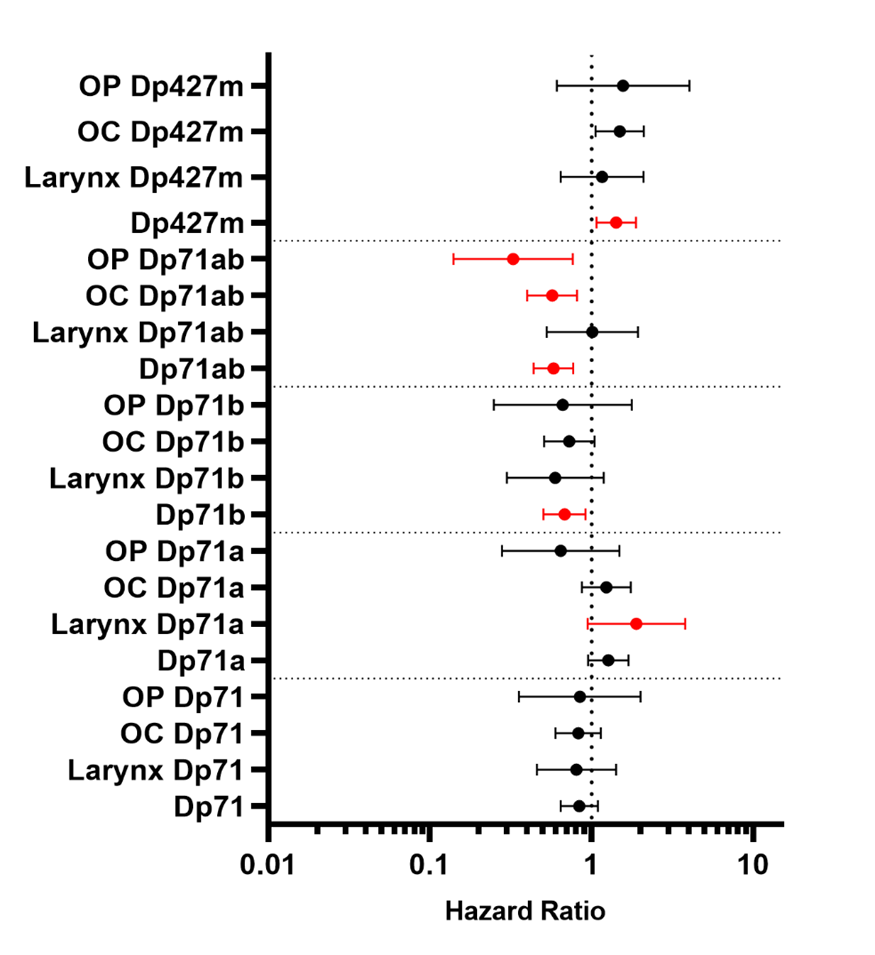
**

Supplementary figure 3. Forest plots showing univariate hazard ratios and 95% confidence intervals.

Red bars indicate significant p values (<0.05) determined using a log rank test. HNSCC total cohort (n=526), larynx (n=117), oral cavity (n=318) and oropharynx (n=81). A hazard ratio of less than 1 suggests high expression is protective and a hazard ratio of more than 1 indicates high expression is a hazard.

**Supplementary table 2.** HNSCC tissue cohort characteristics

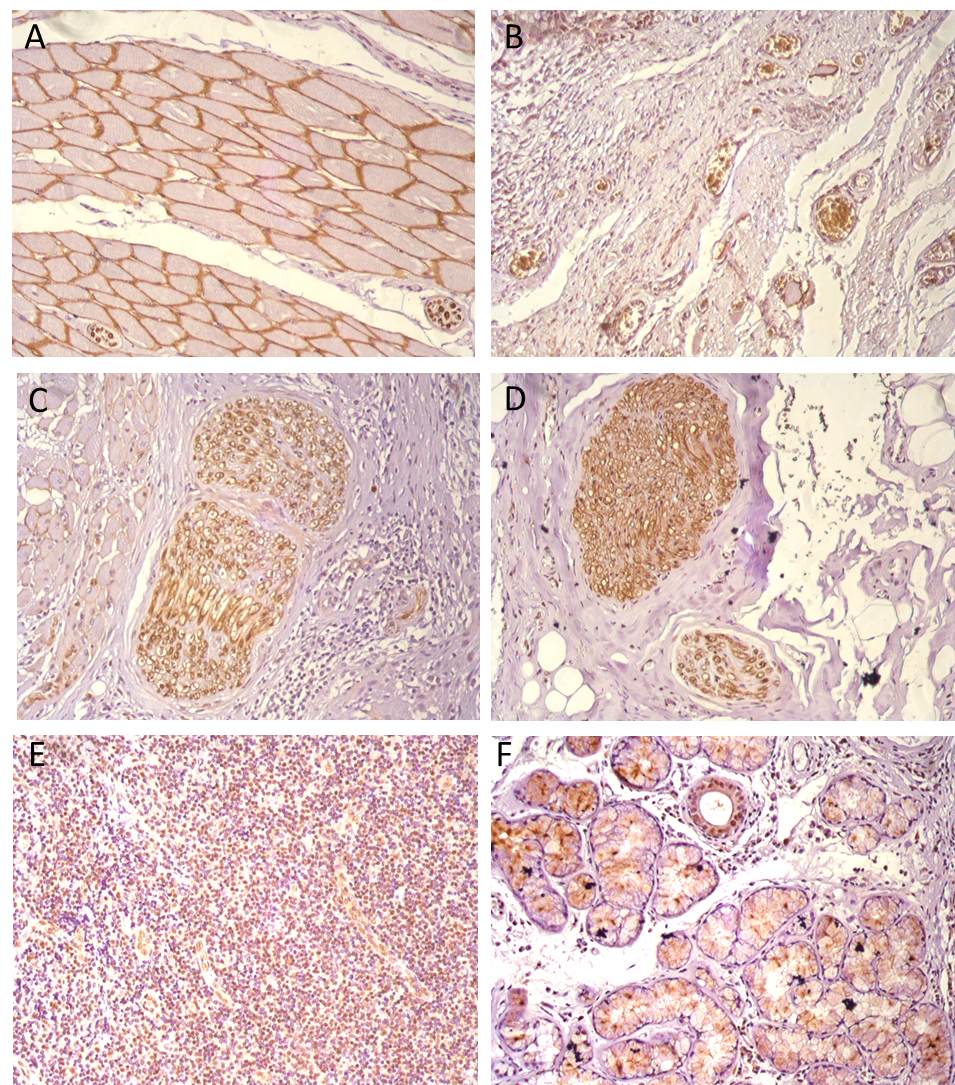


Supplementary figure 4. Immunohistochemistry for dystrophin in non-malignant oral cavity and oropharyngeal tissue (n=50). Representative images from (A) muscle fibres, (B) endothelial cells surrounding blood vessels, (C) salivary gland, (D) nerve cell (top) salivary gland (bottom) (E) lymphocytes, (F) lobules of mucinous salivary glands. All images were taken at x20 magnification.

B

A


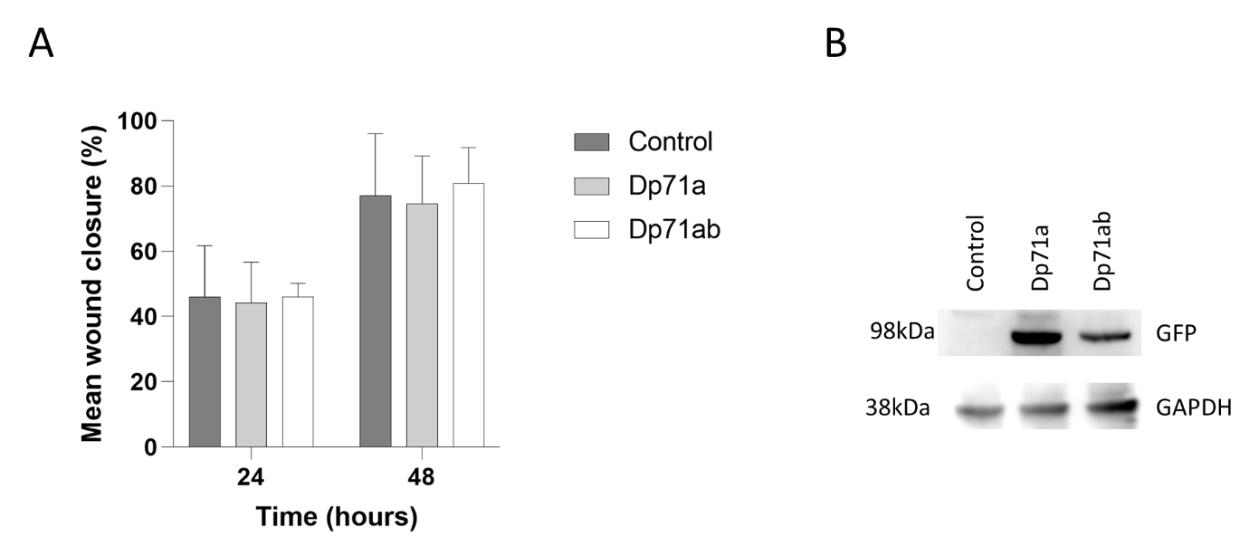


D

C


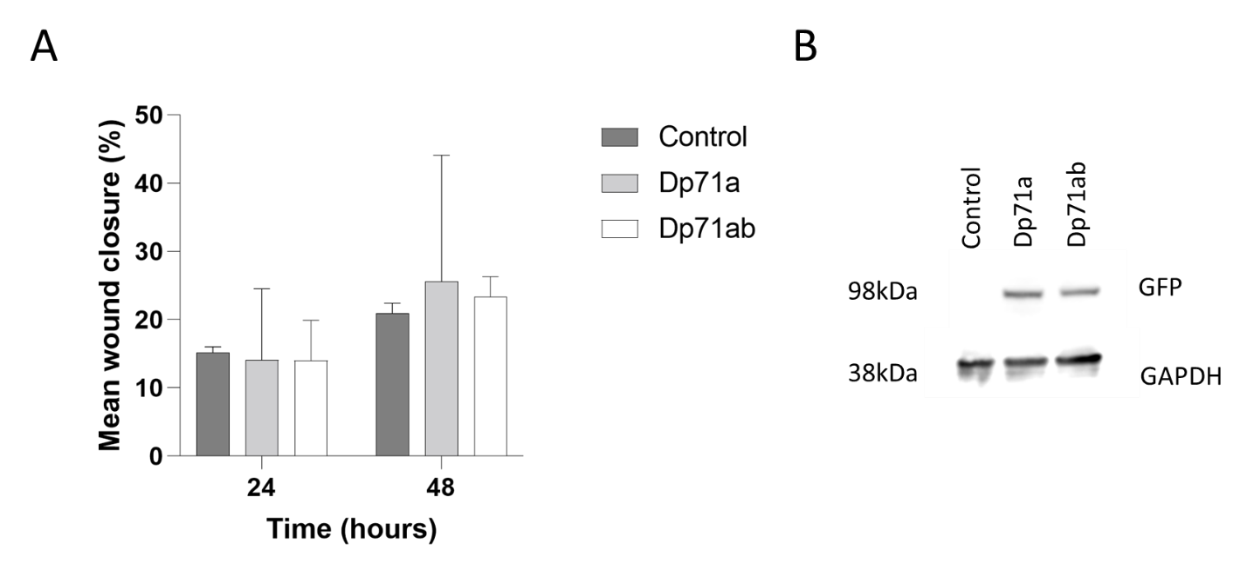


Supplementary figure 5. Mean wound closure (±SD) is shown at 24 and 48 hours for FaDu (A-B) and H314 (C-D) cells. Images (three regions/well) were taken at zero, 24-, and 48-hours and the mean wound closure calculated using Image J. Western blots show confirmation of transfection of Dp71a and Dp71ab constructs using GFP antibody with GAPDH as control. P values were determined using a one-way ANOVA and Dunnett's multiple comparisons test, all results were non-significant. n=3.


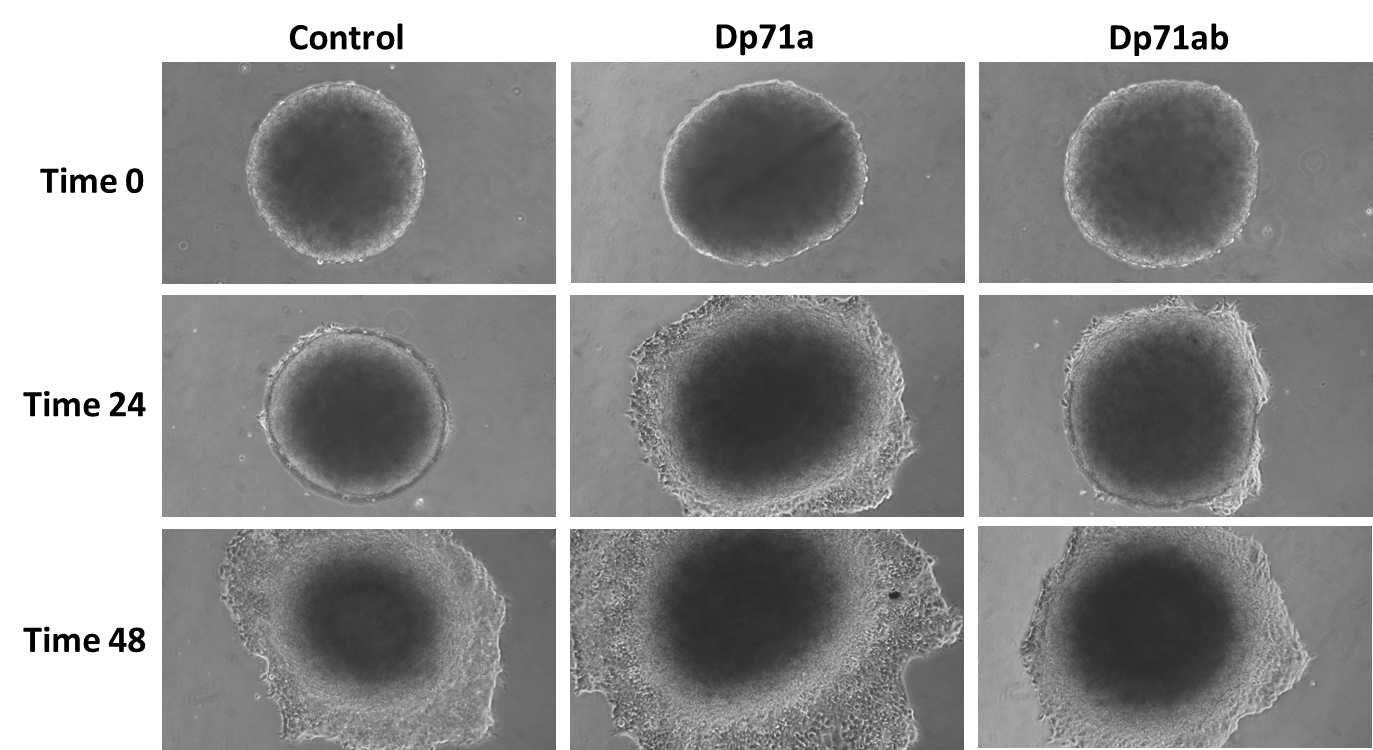


*
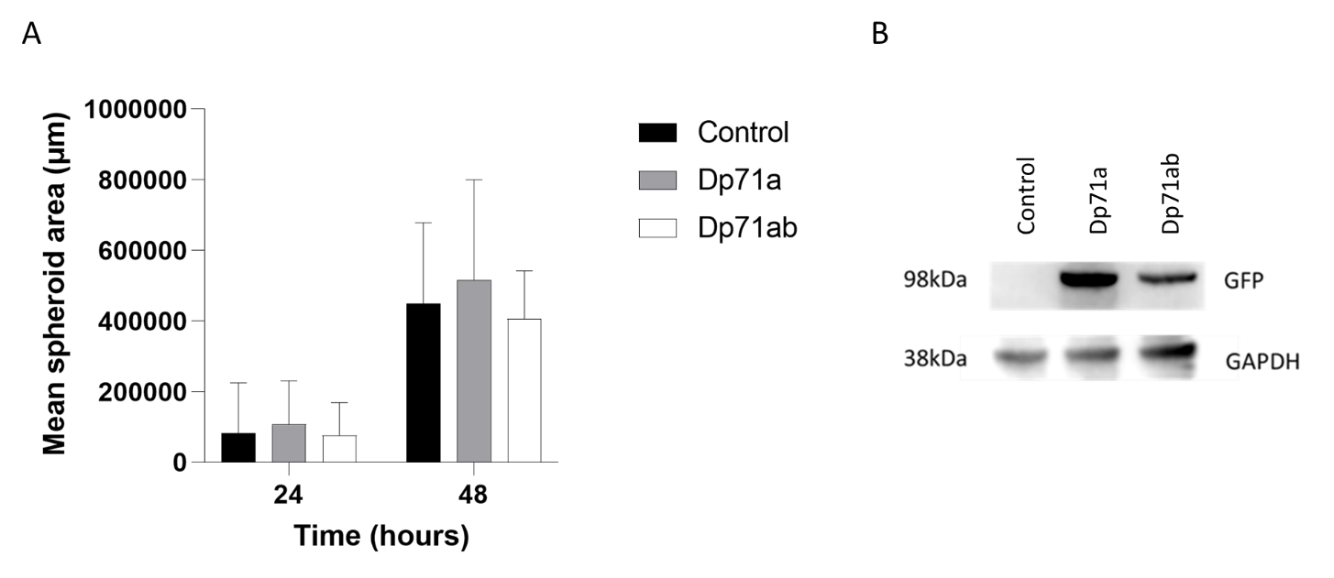
*

Supplementary figure 6. Representative images of FaDu 3D spheroids generated using the hanging drop method. The bar chart displays the mean total spheroid area (±SD) imaged at 24 and 48 hours for the FaDu cell line. The western blot shows confirmation of transfection of Dp71a and Dp71ab constructs using GFP antibody with GAPDH as control (same transfection/blot as per Supplementary Figure 5B since both assays were often conducted in parallel from the same batch of transfected cells). P values were determined using a one-way ANOVA and Dunnett's multiple comparisons test, all results were non-significant. n=3. We were unable to form 3D spheroids with the H314 cell line.

**
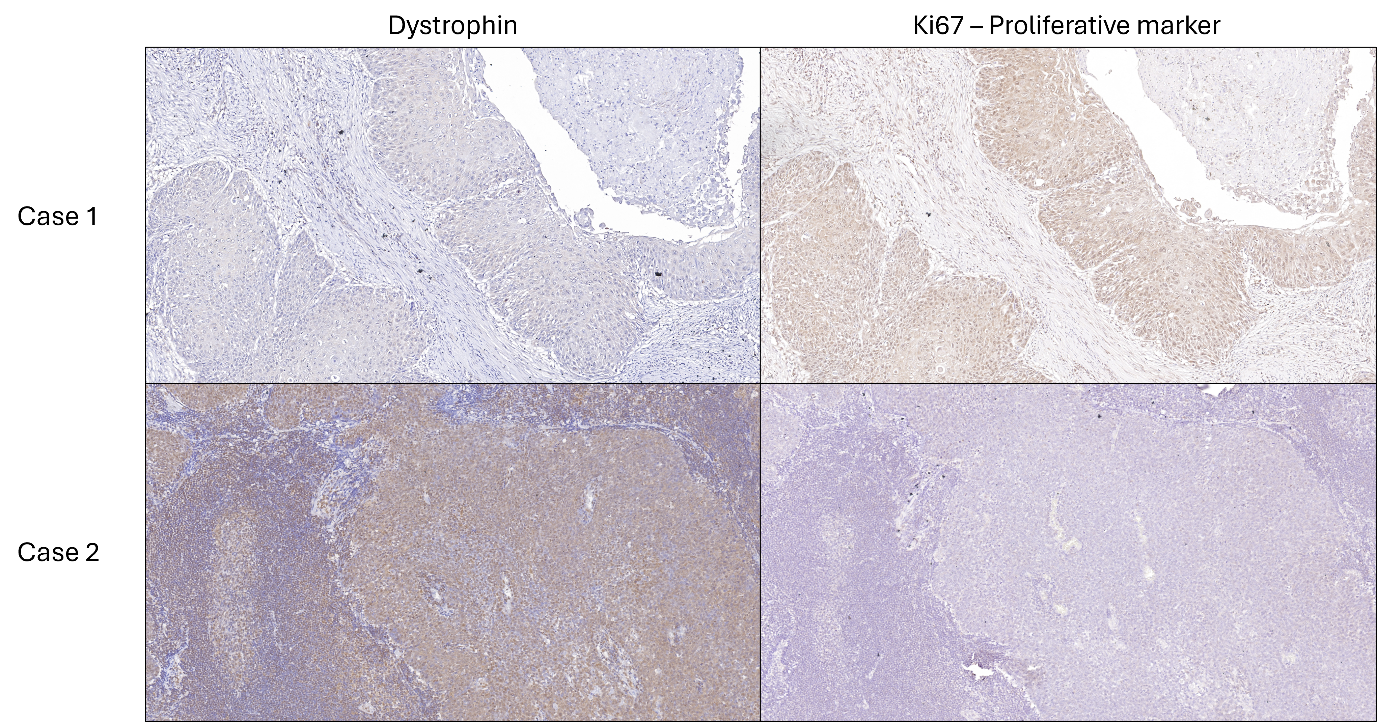
**

B

A

**Supplementary figure 7.** Representative images of HNSCC tissue sections stained with a dystrophin or Ki67 antibody. Case 1 shows low dystrophin and high Ki67 and case 2 shows high dystrophin and low Ki67. The Ki67 proliferative index for each tumour section was calculated blind and cases were subsequently stratified into high and low dystrophin expressing groups. Unpaired t-tests for (A) nuclear dystrophin and (B) nuclear and cytoplasmic dystrophin were non-significant (nuclear p value = 0.0924, nuclear and cytoplasmic p value = 0.6411).

B

A


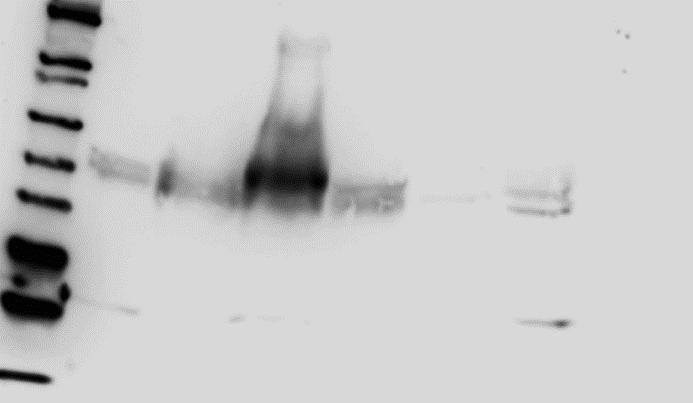

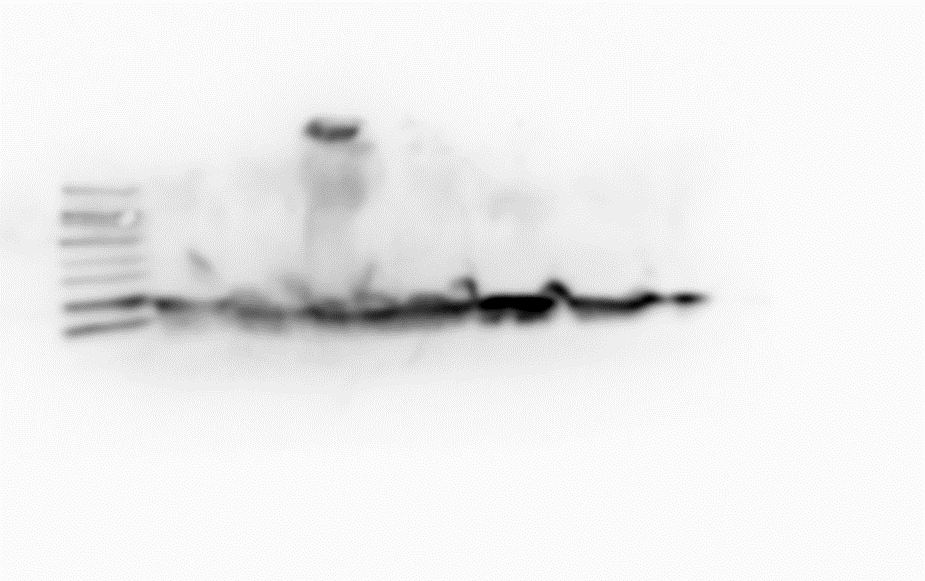


**Supplementary Figure 8.** A. Uncropped dystrophin blot from Figure 4a. B. Uncropped GAPDH blot from Figure 4a.

**
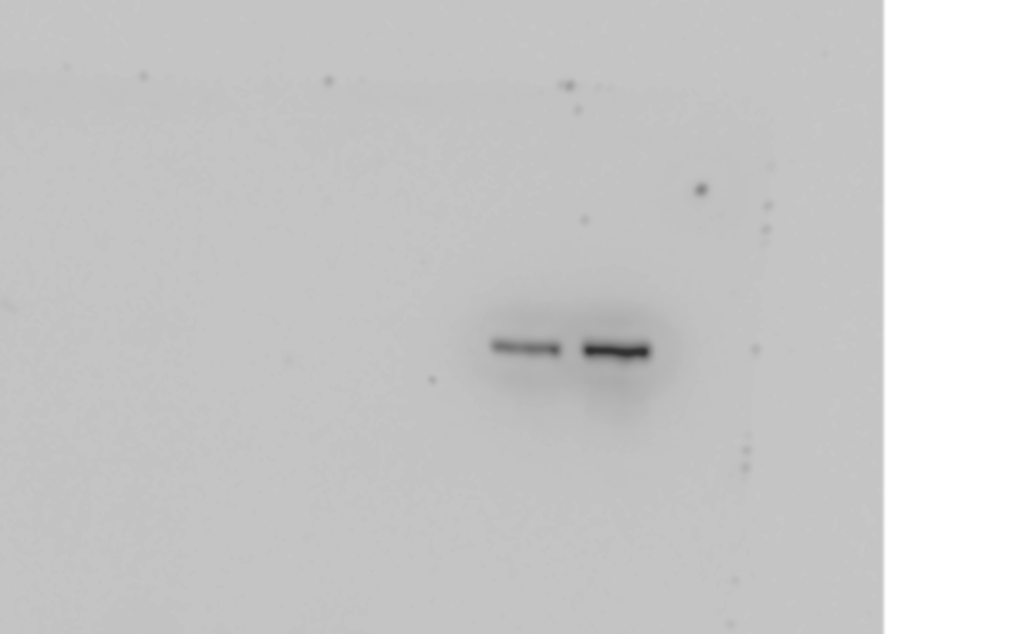
**
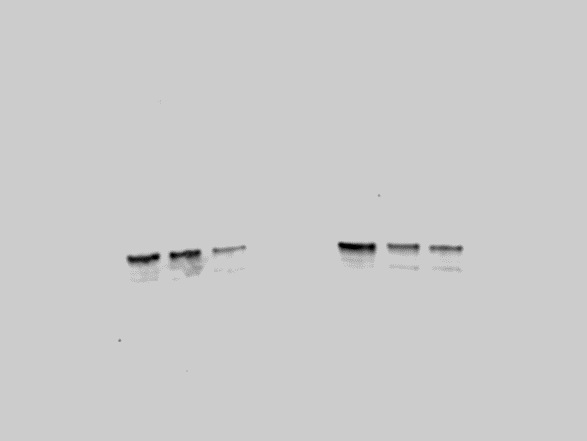

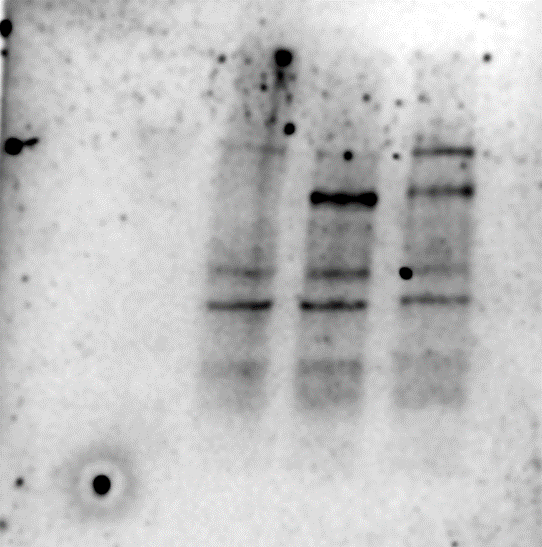


C

B

A

**Supplementary Figure 9.** A. Uncropped GFP blot from Figure 6b. B. Uncropped GFP blot from Figure 6c. C. Uncropped GAPDH blots from Figure 6b and 6c (these samples were run on the same gel).


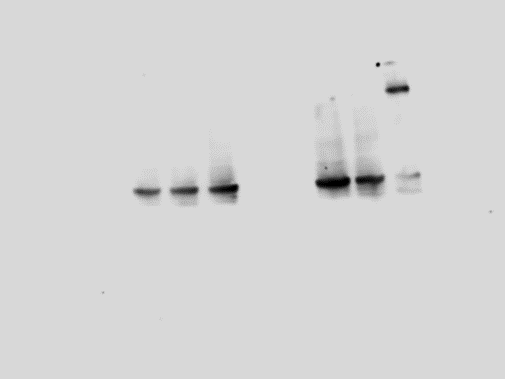

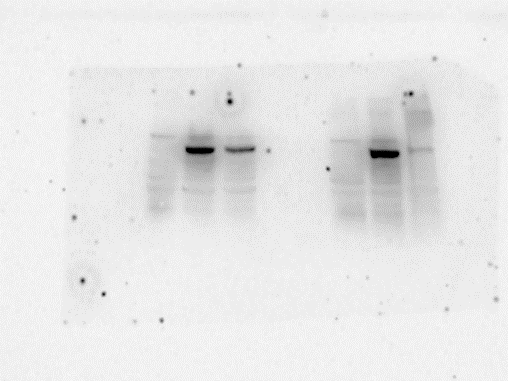


B

A

C


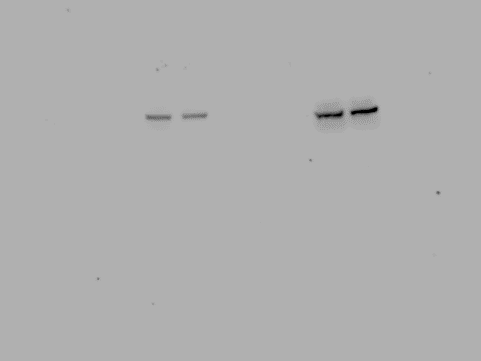

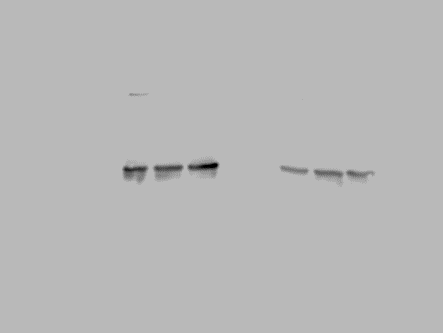


D

Supplementary Figure 10. A. Uncropped GFP blot (left hand side lanes) from Supplementary Figure 5B and Supplementary Figure 6. B. Uncropped GAPDH blot (left hand side lanes) from Supplementary Figure 5B and Supplementary Figure 6. C. Uncropped GFP (left hand side lanes) blot from Supplementary Figure 5D. D. Uncropped GAPDH blot (left hand side lanes) from Supplementary Figure 5D.
